# Antioxidant Defense, Oxidative Modification, and Salivary Gland Function in an Early Phase of Cerulein Pancreatitis

**DOI:** 10.1155/2019/8403578

**Published:** 2019-03-10

**Authors:** Mateusz Maciejczyk, Anna Skutnik-Radziszewska, Izabela Zieniewska, Jan Matczuk, Emilia Domel, Danuta Waszkiel, Małgorzata Żendzian-Piotrowska, Izabela Szarmach, Anna Zalewska

**Affiliations:** ^1^Department of Physiology, Medical University of Bialystok, Poland; ^2^Doctoral Studies, Medical University of Bialystok, Poland; ^3^Cross-sectoral Doctoral Studies, Medical University of Bialystok, Poland; ^4^County Veterinary Inspection, Epidemiology Postgraduate Studies, Medical University of Bialystok, Bialystok, Poland; ^5^Conservative Dentistry Department, Medical University in Bialystok, Poland; ^6^Department of Hygiene, Epidemiology and Ergonomics, Medical University of Bialystok, Poland; ^7^Department of Orthodontics, Medical University of Bialystok, Poland

## Abstract

Acute pancreatitis (AP) is a multifactorial disease characterized by necroinflammatory changes of the pancreas. Our study is the first study which evaluated the relationship between the free radical production, enzymatic and nonenzymatic antioxidants, oxidative damage, and secretory function of the salivary glands of AP rats. Male Wistar rats were divided equally into 2 groups: control (*n* = 9) and AP (*n* = 9). AP was induced by intraperitoneal injection with cerulein and confirmed by higher serum amylase and lipase. We have demonstrated that the superoxide dismutase and glutathione reductase activities, as well as reduced glutathione concentration, were significantly decreased in both the parotid and submandibular glands of AP rats as compared to the control rats. The production of free radicals evidenced as dichlorodihydrofluorescein assay and the activity of NADPH oxidase and xanthine oxidase and IL-1*β* concentration were significantly higher in the parotid and submandibular glands of AP rats compared to the controls. In AP rats, we also showed a statistical increase in oxidation modification products (advanced glycation end products and advanced oxidation protein products), salivary amylase activity, and significant decrease in the total protein content. However, we did not show apoptosis and any morphological changes in the histological examination of the salivary glands of AP rats. To sum up, cerulein-induced AP intensifies production of oxygen free radicals, impairs the redox balance of the salivary glands, and is responsible for higher oxidative damage to these glands. Interestingly, oxidative modification of proteins and dysfunction of the antioxidant barrier are more pronounced in the submandibular glands of AP rats.

## 1. Introduction

Acute pancreatitis (AP) is a multifactorial disease in the course of which digestive enzymes and numerous proinflammatory cytokines are released into the pancreatic interstitium and general circulation, posing a risk of colliquative necrosis of the adjacent tissues and sometimes also distant organs. AP occurs incidentally or recurrently; the effects of the disease may be transient or persistent. The incidence of acute pancreatitis ranges from 10–80,000 to 100,000 per year and the total mortality rate among the hospitalized patients is about 10% [1].

So far, the pathogenesis of acute pancreatitis has not been fully explained, although one of its factors is believed to be oxidative stress (OS) [2]. OS is a situation in which temporarily or chronically increased concentration of reactive oxygen species (ROS) occurs simultaneously with a shortage of ROS inactivation systems, i.e., antioxidative systems [3]. It has been demonstrated that nonneutralized ROS are involved in the initiation and can act as a molecular trigger of acute pancreatitis [4]. ROS lead to oxidative modification of cell membranes and trigger the accumulation of neutrophils and their adhesion to vascular walls. ROS are the main contributor to cytokine synthesis in pancreatic cells, which occurs through direct activation of the oxidant-sensitive transcription factor, NF-*κ*B, which may disturb the integrity of cytoskeleton and initiate mitochondrial dysfunction in pancreatic cells [4–6]. It is therefore believed that ROS play a major role in the consolidation of acute pancreatitis and the development of extrapancreatitic complication [7]. In acute pancreatitis patients, significantly elevated concentration of plasma lipid peroxidation products, decreased concentration of vitamin C, and correlation between oxidative stress markers and severity of the disease have been demonstrated [8, 9].

The pancreas and salivary glands have a similar anatomical structure as a consequence of their analogous embryonic development. It has been demonstrated that the pancreas and salivary glands are formed thanks to a controlled sequence of epithelial-mesenchymal mutual interactions. Also, both these types of glands serve a comparable function: they produce fluid rich in bicarbonates containing digestive enzymes and other ingredients to be delivered into the gut [10]. Moreover, similar mechanisms and structures of the pancreas and the salivary glands are activated in the course of their chronic diseases. It has been demonstrated that alcohol abuse affects the pancreas and salivary glands similarly. Salivary gland dysfunction is believed to be a possible result of the influence of alcohol on antioxidant systems in patients with chronic alcoholic pancreatitis [11]. In the case of chronic pancreatitis caused by immunoaggression, salivary gland damage is considered to be the result of aggressive inflammation associated with lymphocytic infiltration, intensified NADPH oxidase (NOX) activity, and increased production of ROS [10].

Given that saliva produced in the salivary glands plays an important role in the maintenance of oral health and physiology and—due to its highly effective antioxidative properties—provides the first line of antioxidant defense of the gastrointestinal tract against ROS, it appears advisable to evaluate the secretory function, oxidative stress parameters, and antioxidative defense of the salivary glands in the course of acute pancreatitis.

## 2. Materials and Methods

### 2.1. Animals

The protocol of the study was approved by the Local Committee on the Ethical Use of Animals at the Medical University of Bialystok, Poland.

Male Wistar rats (*n* = 18) were obtained from a licensed breeder (8 weeks of initial age, 200–250 g of initial body weight). The animals were provided with standard laboratory conditions: stable temperature (20–21°C ± 2°C), humidity, and 12/12-hour light-dark cycle, as well as unrestricted access to food (standard laboratory rat chow: Agropol, Motycz, Poland) and drinking water. After 7 days of the acclimatization, the rats were randomly divided into 2 equally numbered groups:

1. Control (C) (*n* = 9)
2. Acute pancreatitis (AP) (*n* = 9)

Acute pancreatitis was induced by 2 intraperitoneal cerulein injections (i.p.) (50 *μ*g/kg BW; Sigma-Aldrich, USA) administered with 1-hour interval [12]. Cerulein was dissolved in 1 mL of saline solution and administered by a qualified laboratory staff member. Animals from the control group only received 1 mL of saline solution (i.p.). 24 hours after the cerulein injection, AP condition was confirmed by measuring the activities of serum amylase and lipase. At the end of the experiment (after an overnight fasting), the rats were weighed and anesthetized using phenobarbital (80 mg/kg BW, i.p.).

### 2.2. Salivary Flow Rate

To measure the salivary flow rate, sleeping rats were placed on a heating couch (37°C), and their unstimulated saliva secretion was determined using a preweighed cotton ball. The sterile ball was placed at the bottom of the oral cavity, and the volume of the resulting saliva was calculated by subtracting the weight of the dry cotton ball from the weight of the salivated ball after 15 minutes. It was assumed that 1 mg of saliva is equal to 1 *μ*L [13]. The stimulated saliva secretion was induced by pilocarpine hydrochloride (5 mg/kg BW, i.p.; Sigma-Aldrich, USA). The measurement of the stimulated salivary flow was performed similarly to unstimulated salivary secretion, but 5 minutes after the pilocarpine administration. The measurement lasted 5 minutes [14]. The salivary flow rate was calculated by dividing the volume of saliva by the time necessary for its secretion.

### 2.3. Tissue Preparation

The concentration of tail blood glucose was assessed using glucometer (Accu-Chek, Roche), and then blood samples were collected from the abdominal aorta. Half of the blood volume was poured into a glass tube (to obtain serum), and the other half into a glass tube with heparin (to obtain plasma). All samples were centrifuged (10 min, 4°C, 3000×g). Butylated hydroxytoluene BHT (5 *μ*L 0.5 M BHT in acetonitrile/0.5 mL sample; Sigma-Aldrich, Germany) and protease inhibitor (Complete Mini, Roche, France) were added to the resulting supernatants [14]. The samples were precooled in liquid nitrogen and stored at -80°C until needed for assays.

Next, the left parotid and submandibular glands were collected by a qualified laboratory staff member. The salivary gland tissue was immediately washed, dried and weighed, freeze-clamped with aluminum tongs, frozen in liquid nitrogen, and stored at -80°C until used [15]. The right salivary glands were submerged in 10% formalin and then processed for paraffin embedding.

### 2.4. Preparation of Homogenates

On the day of the biochemical analysis, the salivary glands were rinsed and diluted 1 : 10 (*w*/*v*) in ice-cold phosphate-buffered saline (PBS; 0.02 M, pH 7.0). In order to prevent sample oxidation and proteolysis, BHT and proteolysis inhibitor were added. The tissues were homogenized with a glass homogenizer (5000 rpm/L min, on ice; Omni TH, Omni International, USA), and the resulting suspensions were sonicated with an ultrasonic cell disrupter (1800 J, 3 × 20 s, on ice; UP 400S, Hielscher, Germany). The homogenates were centrifuged (20 min, 4°C, 3000×g) for supernatant collection and immediate assay [16–18].

### 2.5. Biochemical Analysis

Antioxidants and oxidative damage products were determined both in homogenates of the parotid and submandibular glands and plasma/blood serum. All reagents for the biochemical assays (unless otherwise specified) were obtained from the Sigma-Aldrich, Germany/USA. The absorbance and fluorescence were measured using Infinite M200 PRO Multimode Microplate Reader (Tecan). All determinations (with the exception of SOD, CAT, TAC, and TOS) were determined in duplicate samples and standardized to mg of total protein.

### 2.6. Antioxidant Assays

Cu-Zn superoxide dismutase (SOD, E.C. 1.15.1.1) activity was analyzed spectrophotometrically in triplicate samples by measuring the inhibition rate of adrenaline oxidation to adrenochrome [19]. The absorbance was evaluated at 480 nm wavelength. 1 unit of SOD activity was defined as the quantity of the enzyme to inhibit the oxidation of adrenaline by 50%.

Catalase (CAT, E.C. 1.11.1.6) activity was determined spectrophotometrically in triplicate samples by measuring the decomposition rate of hydrogen peroxide at 240 nm wavelength [20]. 1 unit of CAT activity was defined as the amount of the enzyme needed to decompose 1 mmol hydrogen peroxide per 1 minute.

Salivary peroxidase (SP, E.C. 1.11.1.7) activity was assayed spectrophotometrically according to Mansson-Rahemtulla et al. [21]. This method is based on the reduction of 5,5′-dithio-bis-(2-nitrobenzoic acid) (DTNB) to trinitrobenzene acid. The absorbance of the resulting complex was measured at 412 nm wavelength (5 times at 30 s intervals).

Serum glutathione peroxidase (GPx, E.C. 1.11.1.9) activity was determined spectrophotometrically according to Paglia and Valentine [22], which is a method based on the reduction of organic peroxides in the presence of NADPH. The absorbance was measured at 340 nm wavelength. 1 unit of GPx activity was defined as the amount of the enzyme to catalyze the oxidation of 1 *μ*mol of NADPH for 1 minute.

Glutathione reductase (GR, E.C. 1.8.1.7) activity was estimated spectrophotometrically by measuring the decrease in NADPH absorbance at 340 nm wavelength [23]. 1 unit of GR activity was defined as the quantity of the enzyme to catalyze the oxidation of 1 *μ*mol of NADPH for 1 minute.

Reduced glutathione (GSH) level was measured spectrophotometrically based on the reaction of GSH with DTNB according to Moron et al. [24]. The absorbance of the resulting complex was evaluated at 412 nm wavelength.

Total antioxidant capacity (TAC) was assayed spectrophotometrically at 660 nm wavelength based on the reaction of 2,2-azino-bis(3-ethylbenzothiazoline-6-sulfonic acid) radical cation (ABTS^∗^^+^) with antioxidants contained in the samples [25]. TAC levels were measured in triplicate samples and calculated from the calibration curve for Trolox (6-hydroxy-2,5,7,8-tetramethylchroman-2-carboxylic acid).

### 2.7. ROS Generation

NADPH oxidase (NOX, E.C. 1.6.3.1) activity was analyzed by luminescence assay using lucigenin as a luminophore [26]. NOX-mediated superoxide radical (O_2_^−^) production was determined by measuring lucigenin luminescence at 30°C every 5 s for 10 min. One unit of NOX activity was defined as the quantity of enzyme required to release 1 nmol of O_2_^−^ for 1 minute.

Xanthine oxidase (XO, E.C. 1.17.3.2.) activity was estimated by uric acid (UA) production from xanthine by measuring the increase in UA absorbance at 290 nm [27]. The XO activity was provided by the difference between the rate in the complete reaction mixture and that in the blank (reaction mixture without xanthine). One unit of XO activity was defined as the amount of enzyme required to release 1 *μ*mol of UA for 1 minute.

ROS production was measured fluorimetrically using 2,7-dichlorodihydrofluorescein diacetate (DCFH-DA). DCFH-DA is deesterified within cells to 2,7-dichlorodihydrofluorescein (DCFH), which is capable to oxidation by oxygen free radicals [28]. Oxidized DCFH fluoresces at 488/525 nm and its intensity is used to calculate the rate of ROS production. ROS production rate was calculated from the calibration curve for DCFH and expressed as nmol/h/mg protein.

NOX, XO, and DCFH assays were performed immediately after sample collection [15].

### 2.8. Oxidative Damage Assays

Advanced glycation end product (AGE) content was analyzed spectrofluorimetrically by measuring AGE-specific fluorescence [29, 30]. Samples were diluted 1 : 50 (*v*/*v*) in PBS (0.02 M, pH 7.0), and the fluorescence intensity was measured at 440/370 nm in a 96-well microplate spectrophotometer. The fluorescence of AGE was expressed as arbitrary fluorescence units (AFU)/mg protein.

Advanced oxidation protein product (AOPP) concentration was estimated spectrophotometrically at 340 nm wavelength by measuring the oxidative capacity of the iodine ion [29]. For plasma AOPP determination, samples were diluted 1 : 50 (*v*/*v*) in PBS.

Total oxidant status (TOS) levels were assayed bichromatically (560/800 nm) based on the oxidation of Fe^2+^ to Fe^3+^ in the presence of the oxidants contained in the sample [31]. TOS was measured in triplicate samples and expressed as micromolar hydrogen peroxide equivalent per liter.

Oxidative stress index (OSI) was calculated by dividing TOS by TAC values and expressed in % [32].

### 2.9. Salivary Gland Inflammation and Apoptosis

The concentration of interleukin-1*β* (IL-1*β*) was measured using commercial ELISA kit (R&D Systems, Canada, Minneapolis, USA) in accordance to the manufacturer's instructions. The absorbance was analyzed at 450 nm.

Caspase-3 (CAS-3, EC 3.4.22.56) activity was determined colorimetrically using Ac-Asp-Glu-Val-Asp-p-nitroanalide as a substrate [33]. The amount of p-nitroaniline (pNA) released by CAS-3 activity was quantitated by measuring the absorbance at 405 nm.

### 2.10. Lysosomal Exoglycosidases

The activity of lysosomal exoglycosidases (N-acetyl-*β*-hexosaminidase (HEX, E.C. 3.2.1.52) and *β*-glucuronidase (GLU, E.C. 3.2.1.31)) was measured colorimetrically by the method of Marciniak et al. (HEX) [34] and Chojnowska et al. (GLU) [35]. 4-Nitrophenyl-N-acetyl-*β*-glucosamide and 4-nitrophenyl-*β*-D-glucuronide (Sigma, St. Louis, MO, USA) was used to evaluate HEX/GLU activity, respectively. The absorbance of released 4-nitrophenol was measured at 405 nm.

### 2.11. Protein Assay

The content of total protein was estimated using the bicinchoninic acid (BCA) method with bovine serum albumin (BSA) as a standard. A commercial kit was used according to the manufacturer's instructions (Thermo Scientific PIERCE BCA Protein Assay) (Rockford, IL, USA).

The activity of salivary amylase (SA, EC 3.2.1.1) was determined colorimetrically using 3,5-dinitrosalicylic acid as a substrate. A standard curve was made for maltose in the concentration range of 0–180 mg/mL. The analyses were performed in triplicate samples [36].

### 2.12. Histology

The salivary glands, placed in 10% formalin, were cut in five micron sections and processed for hematoxylin-eosin staining. The sections were observed under light microscope (Olympus BX51, Olympus) and analyzed by a histologist at a magnification 60x.

### 2.13. Statistical Analysis

The data were processed using GraphPad Prism 7 (GraphPad Software, La Jolla, USA) and Statistica 12.0 (StatSoft, Cracow, Poland). The Kolmogorov-Smirnov test showed normal distribution of the obtained results, which was the reason for using parametric analysis. The statistical significance was defined as *p* ≤ 0.05. Unpaired Student's *t*-test and Pearson's correlation method were used. The results were expressed as mean ± SD. The sample size was set based on a previously conducted pilot study (the power of the test was set at 0.9).

## 3. Results

### 3.1. General Characteristics

AP rats showed significantly higher activity of serum amylase (*p* < 0.0001) as well as lipase (*p* < 0.0001) as compared to the control groups. Moreover, we observed more than 4 times higher increment of CRP concentration in the serum of AP rats compared to the controls (*p* = 0.001). Furthermore, serum glucose in the AP group was also significantly raised in comparison with the control rats (*p* < 0.05). It should also be mentioned than one rat of the AP group died after an injection of cerulein (Table 1).

**Figure 1:**
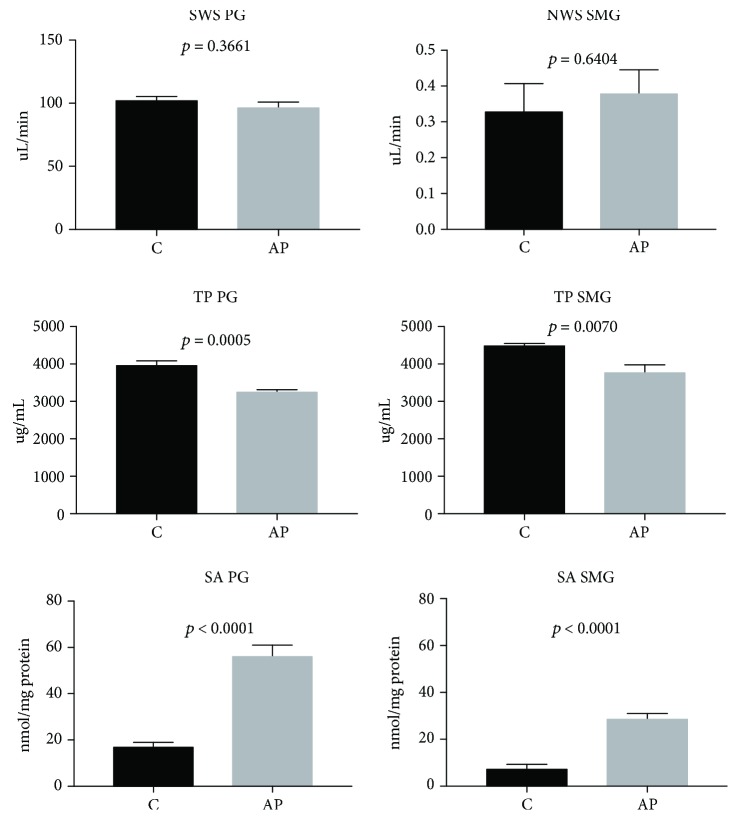
The effect of cerulein-induced AP on unstimulated and stimulated saliva secretion, protein concentration, and salivary amylase activity (C: controls; AP: acute pancreatitis; SWS: stimulated saliva secretion; NWS: nonstimulated saliva secretion; TP: total protein; SA: salivary amylase; PG: parotid glands; SMG: submandibular glands).

The parotid and submandibular gland weight did not differ between the examined and the control group (Table 1). The unstimulated and stimulated salivary flow did not differ between the both groups either (Figure 1).

We noticed that the activity of salivary amylase in both the submandibular (*p* < 0.05) and parotid glands (*p* < 0.05) of AP rats was considerably higher than in the controls. The concentration of protein in the homogenates of the submandibular (*p* < 0.05) and parotid (*p* < 0.05) glands of rats from the AP group was significantly lower compared to the salivary glands of the controls (Figure 1).

### 3.2. Serum and Plasma Antioxidants and Oxidative Stress Markers

The effect of cerulein-induced AP on serum and plasma redox balance is presented in Table 2.

We observed a significant 38% increase in CAT activity (*p* < 0.05) with simultaneous 30% decrease of GPx activity (*p* < 0.05) and 28% decrease of GR activity (*p* < 0.05) in serum as well as 34% reduction of GSH concentration (*p* < 0.05) in plasma of AP rats vs. the controls (Table 2). Moreover, we noticed a 22% drop in TAC (*p* < 0.0005) concentration in AP rats' plasma compared to the control group (Table 2).

The production of free radicals evidenced as activity of NOX (*p* < 0.0001), XO (*p* < 0.0001), and dichlorodihydrofluorescein assay (*p* < 0.0001) was significantly higher in the plasma of AP rats compared to the control (Table 2).

We also demonstrated a significant 18% rise of AGE content (*p* < 0.05) as well as 456% increase in AOPP concentration (*p* < 0.0005) in plasma of the AP group compared to the control group (Table 2).

### 3.3. Parotid Gland Antioxidants and Oxidative Stress Markers

**Figure 2:**
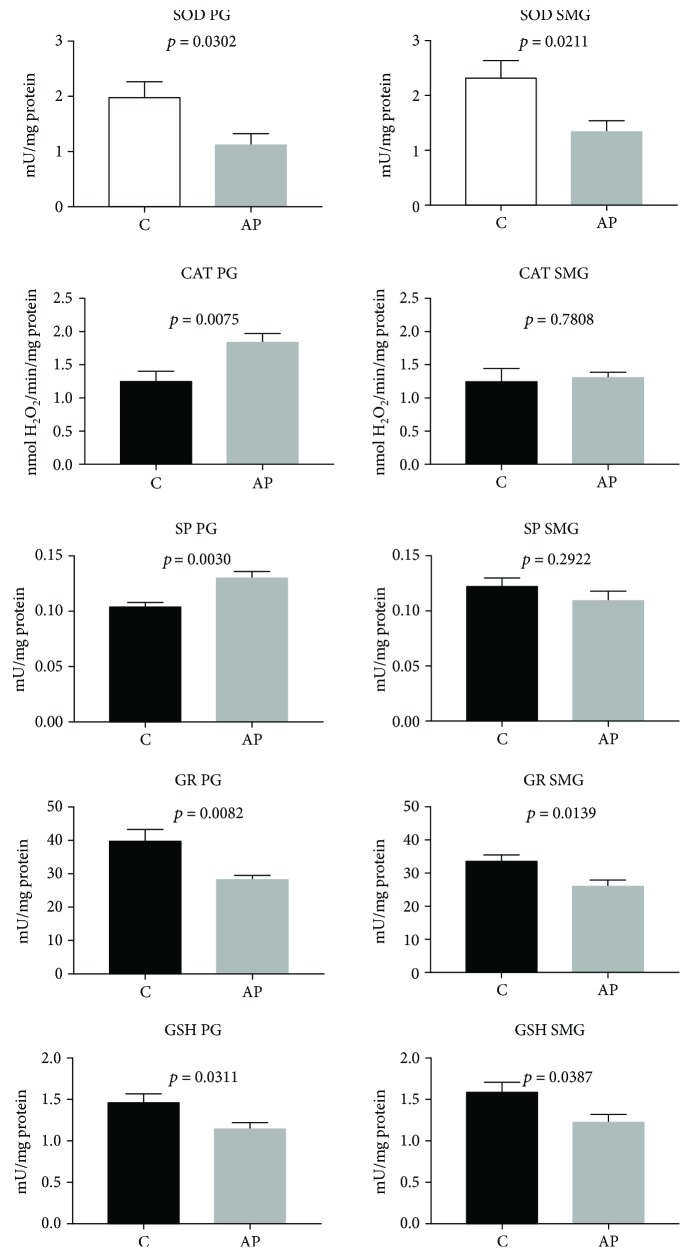
The effect of cerulein-induced AP on salivary gland antioxidant defense mechanisms (C: control; AP: acute pancreatitis; PG: parotid glands; SMG: submandibular glands; SOD: superoxide dismutase; CAT: catalase; SP: salivary peroxidase; GR: glutathione reductase; GSH: reduced glutathione).

**Figure 3:**
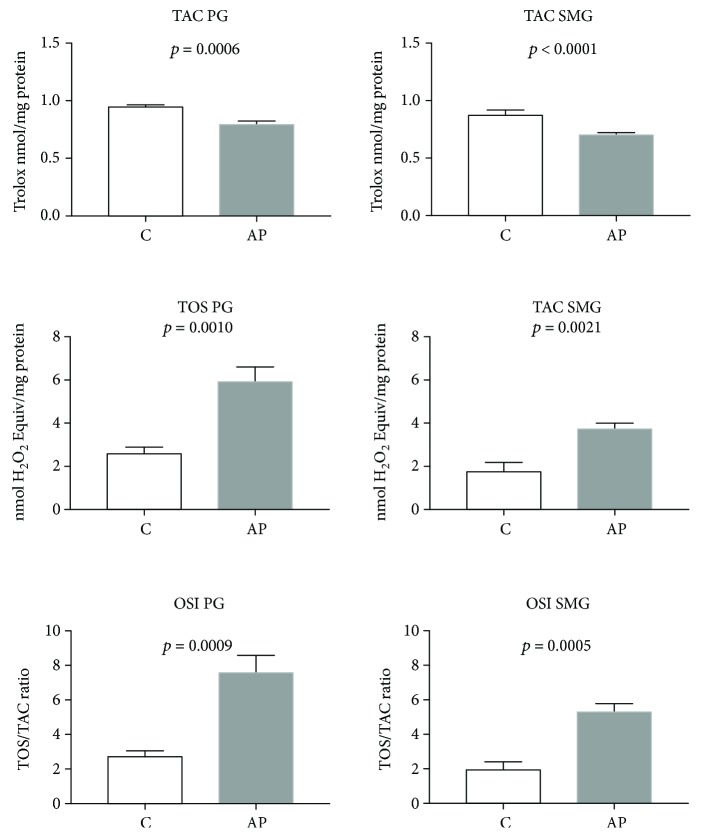
The effect of cerulein-induced AP on salivary gland total antioxidant capacity, total oxidant status, and oxidative status index (C: control; AP: acute pancreatitis; PG: parotid glands; SMG: submandibular glands; TAC: total antioxidant capacity; TOS: total oxidant status; OSI: oxidative status index).

**Figure 4:**
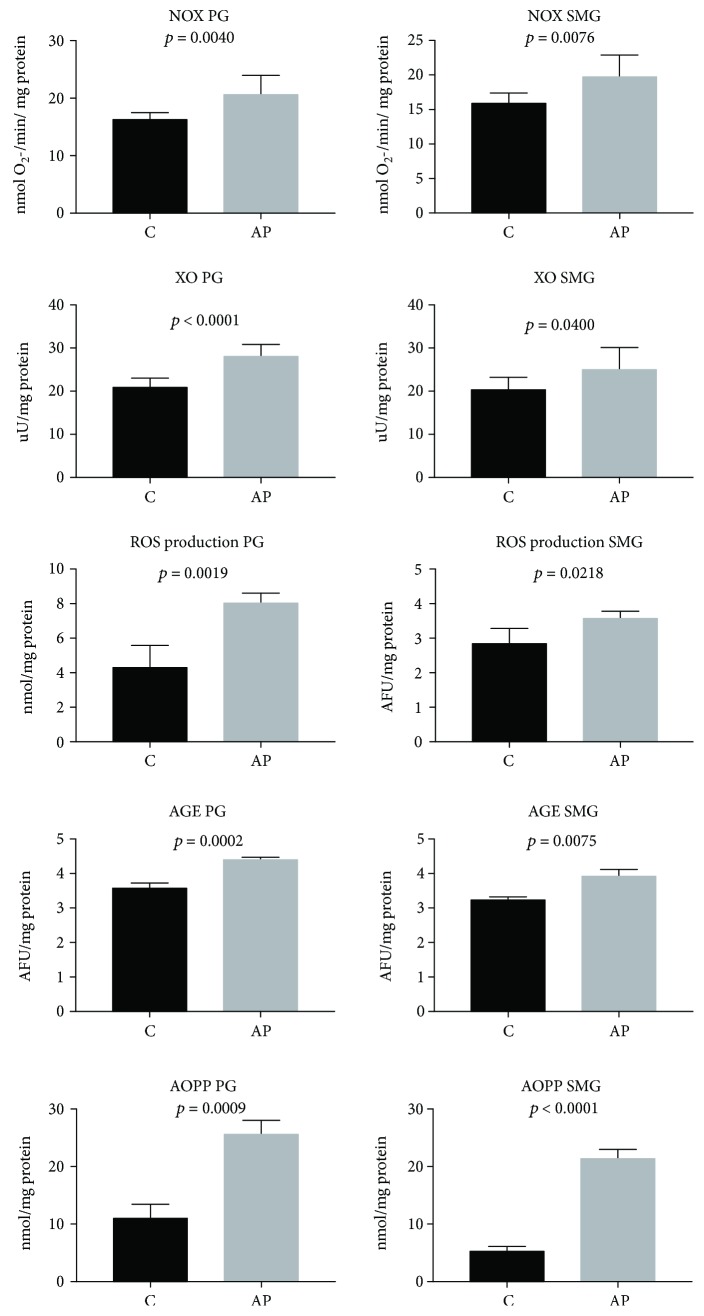
The effect of cerulein-induced AP on free radical generation and protein oxidative modification in the salivary glands of rats (C: control; AP: acute pancreatitis; PG: parotid glands; SMG: submandibular glands; AGE: advanced glycation end products; AOPP: advanced oxidation protein products; NOX: NADPH oxidase; XO: xanthine oxidase; ROS: reactive oxygen species).

The effect of cerulein-induced AP on the parotid gland SOD, CAT, SP, and GR activities as well as GSH, TAC, TOS, OSI, AGE, and AOPP concentrations as well as NOX and XO activities and ROS production is presented in Figures 2–4.

As compared with C rats, the parotid glands of rats from the AP group were characterized by significantly decreased SOD and GR activities (*p* < 0.05 and *p* < 0.0001, respectively) (Figure 2). At the same time, we observed considerably increased activities of CAT and SP (*p* < 0.0001 and *p* < 0.0001, respectively) in the parotid glands of AP rats compared to the controls (Figure 2). We also noticed significantly reduced GSH (*p* < 0.05) (Figure 2) and TAC as well as increased TOS concentrations and raised OSI (*p* < 0.00001, *p* < 0.005, and *p* < 0.00001, respectively) in the parotid glands of AP rats as compared to the control group (Figure 3).

Moreover, the parotid glands of AP rats were characterized by a significant increment in ROS production (*p* < 0.002), NOX (*p* < 0.005), and XO activity (*p* = 0.001), as well as AGE (*p* < 0.0005) and AOPP (*p* < 0.00001) concentrations as compared to the control (Figure 4).

### 3.4. Submandibular Gland Antioxidants and Oxidative Stress Markers

The effect of cerulein-induced AP on the submandibular gland SOD, GR, CAT, and SP activities as well as GSH, TAC, TOS, OSI, AGE, and AOPP as well as NOX and XO activities and ROS production concentrations is presented in Figures 2–4.

We noticed that the activity of SOD and GR in the AP submandibular glands was significantly decreased (*p* < 0.05 and *p* < 0.05, respectively) as compared to the controls (Figure 2). GSH (*p* < 0.05) (Figure 2) and TAC (*p* < 0.0001) concentrations were considerably lower, whereas TOS concentration and OSI were significantly higher in the submandibular glands of AP rats as compared to the control group (*p* < 0.005 and *p* < 0.00001, respectively) (Figure 3).

Moreover, the submandibular glands of AP rats were characterized by increased ROS production (*p* < 0.05), NOX (*p* < 0.002), and XO activity (*p* < 0.05), as well as higher concentrations of AGE (*p* < 0.0001) and AOPP (*p* < 0.0001) as compared to the control group (Figure 4).

### 3.5. Salivary Gland Inflammation and Apoptosis

**Figure 5:**
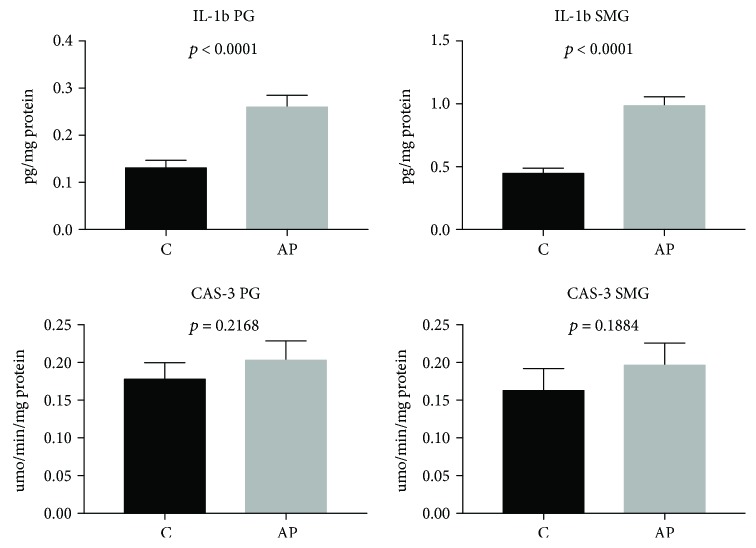
The effect of cerulein-induced AP on salivary gland inflammation and apoptosis (C: control; AP: acute pancreatitis; PG: parotid glands; SMG: submandibular glands; IL-1*β*: interleukin-1*β*; CAS-3: caspase-3).

IL-1*β* concentration was significantly higher in the parotid and submandibular glands of AP rats vs. the control group (*p* < 0.0001). However, CAS-3 activity did not differ significantly between the salivary glands of AP rats and the controls (Figure 5).

### 3.6. Serum and Salivary Exoglycosidases

**Figure 6:**
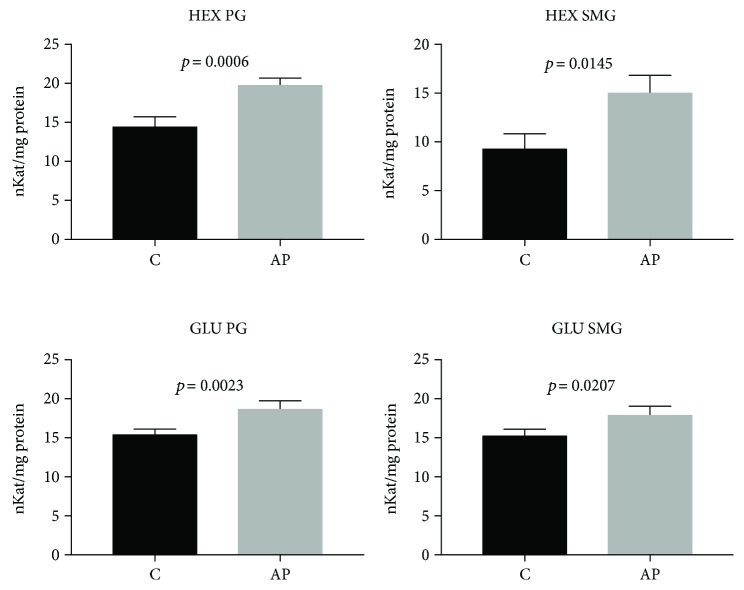
The effect of cerulein-induced AP on the activity of lysosomal exoglycosidases in the salivary glands of rats (C: control; AP: acute pancreatitis; PG: parotid glands; SMG: submandibular glands; HEX: N-acetyl-*β*-hexosaminidase; GLU: *β*-glucuronidase).

Serum HEX and GLU activity did not differ statistically in the group of cerulein-induced AP as compared to the control rats (Table 3). However, the elevated activity of both exoglycosidases we have observed in the parotid (*p* = 0.0006, *p* < 0.005, respectively) and submandibular glands (*p* < 0.02, *p* < 0.03, respectively) of AP rats as compared to the control group (Figure 6).

### 3.7. Correlations

The Spearman rank correlation coefficient showed that TAC concentration in the parotid glands of AP rats was positively correlated with SP activity (*r* = 0.78, *p* = 0.001). In the parotid glands of AP rats, negative correlation was noted between GSH and AOPP concentrations (*r* = -0.49, *p* = 0.03), and in their submandibular glands, GSH was negatively correlated with AGE concentration (*r* = -0.53, *p* = 0.02).

In the parotid and submandibular glands of AP rats, negative correlations were noted between amylase activity and protein concentration (*r* = -0.65, *p* = 0.01 and *r* = -0.51, *p* = 0.02, respectively).

In the parotid and submandibular glands of AP rats, a positive correlation was observed between TAC and GR activity (*r* = 0.67, *p* = 0.02 and *r* = 0.56, *p* = 0.03, respectively), between NOX activity and ROS (*r* = 0.89, *p* = 0.001 and *r* = 0.91, *p* = 0.001, respectively), and between NOX activity and IL-1*β* concentration (*r* = 0.72, *p* = 0.001 and *r* = 0.89, *p* = 0.0005, respectively).

### 3.8. Histological Examination

**Figure 7:**
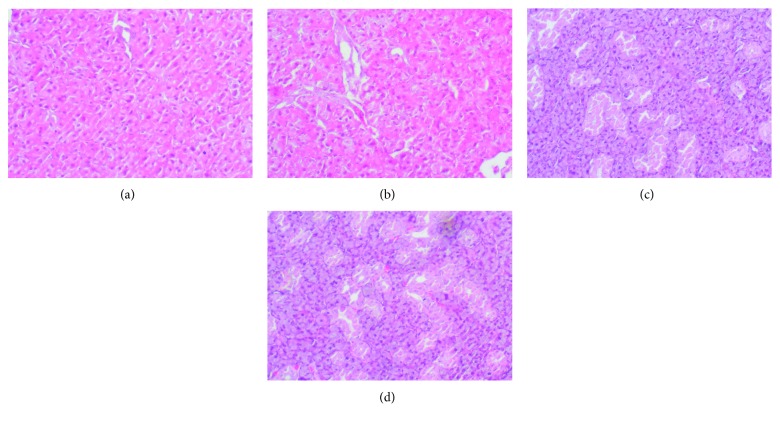
The effect of cerulein-induced AP on histological observation in the salivary glands: (a) parotid gland of the control rats; (b) parotid glands of AP rats; (c) submandibular glands of control rats; (d) submandibular glands of AP rats. Magnification 60x.

We did not show any morphological changes in the histological examination of both salivary glands in AP rats in comparison to the control group. In cerulein-induced AP, both the parotid and the submandibular glands are characterized by the normal structure and normal size of the secretory elements (Figure 7).

## 4. Discussion

This is the first study to evaluate the effect of cerulein-induced AP on redox balance in the salivary glands. It was demonstrated that cerulein-induced acute pancreatitis affects the salivary redox homeostasis and generally downregulates their antioxidant defense mechanisms.

Experimental models are extremely helpful in explaining the pathologies occurring in humans. However, their disadvantage is the fact that the obtained results cannot correspond directly with the functioning of the human body. Nevertheless, considering the high morphological similarity of the human and rat salivary glands—mainly the final secretory products and acinar and ductal system—in our study, we used sexually mature male rats of the Wistar strain [37]. From the available AP experimental models, we chose the one in which acute pancreatitis is induced by a single intraperitoneal injection of cerulein [12]. Cerulein is analogous to cholecystokinin (it causes maximum pancreatic secretion of lipase and amylase), which leads to acinar cell death, edema formation, inflow of inflammatory cells to the pancreas, and inflammation of the organ [38]. In our study, we observed a significant increase in the activity of both pancreatic amylase and lipase, which confirms the development of acute pancreatitis after cerulein admission and is consistent with the earlier results [12]. It is believed that increased CRP concentration is one of the markers of AP; it is therefore not surprising that in our experiment, we noticed significantly elevated CRP concentration, which is consistent with the previously published data [39]. Furthermore, the 10% mortality in the AP group observed by us reflects the overall mortality rate typical of the disease.

The existence and extent of oxidative stress can be evaluated by means of a number of biomarkers, including the measurement of the concentration/activity of individual antioxidants (SOD, CAT, GPx, and GSH) as well as the evaluation of the concentrations of oxidative modification products associated with ROS and their derivatives [40]. The exceptionally useful markers utilized in the assessment of oxidative stress intensity are also total antioxidant capacity (TAC), total oxidative status (TOS), and oxidative stress index (OSI) [41]. It is believed that TAC reflects the efficiency of all, both enzymatic and nonenzymatic, antioxidant defense mechanisms, whereas TOS is the sum of all oxidants present in the sample and OSI evaluates the relationship between total antioxidant mechanisms and oxidant concentration [13].

It is thought that NOX in activated neutrophils is the main source of ROS in the course of cerulein AP, which seems to agree with our results. Cerulein, when combined with its membrane receptor, activates phospholipase C and induces inositol 1,4,5-triphosphate-dependent Ca^2+^ release from endoplasmic reticulum in pancreatic cells. The effect of increased Ca^2+^ concentration is the activation of NADPH oxidase and nitric oxide synthase (iNOS). The stimulated NADPH oxidase provides superoxide anion radical, which could be neutralized or can promote the formation of other, more reactive oxygen species. Inducible iNOX produces nitric oxide, followed by the activation of NF-*κ*B and secretion of proinflammatory cytokines, which initiates inflammation and deepens redox balance disorders [6, 42]. It can also be confirmed by the results of our study, in which we showed a positive correlation between NOX activity and IL-1*β* concentration. It was demonstrated that in the course of AP induced by cerulein, the redox balance is disturbed towards the reaction of oxidation. Both in patients and in experimental studies, there has been a significant increase in the concentration of numerous products of cell component oxidative modifications as well as abnormal antioxidant activity/concentration in various organs and serum [5, 6, 43], as confirmed by our results. We observed significant increases in plasma AGE and AOPP concentrations as well as subnormal levels of most examined antioxidants (with the exception of CAT activity) in serum and plasma of AP rats compared to the controls.

However, not much is known about the influence of cerulein-induced AP on the redox balance and the function of the salivary glands of rats.

As already mentioned, the main ROS produced by NADPH oxidase is superoxide anion radical, which is normally easily converted into hydrogen peroxide with the participation of superoxide dismutase, as well as NO produced as a result of iNOX stimulation. SOD cooperates with other enzymes: catalase, peroxidase, and glutathione peroxidase that catalyze the reaction of converting hydrogen peroxide into water, which enables the preservation of redox balance [44]. For AP cerulein, the observed significant 72% decrease in SOD activity in the parotid gland and a 42% decrease in SOD activity in the submandibular gland resulted in the inability of complete inactivation of the produced superoxide anion radical by interacting antioxidative systems. It should be highlighted that the greatest threat to the cell is the reaction of the nonneutralized superoxide anion radical with nitrogen oxide (NO), which produces highly reactive peroxynitrite. Peroxynitrite, in turn, can react with virtually any encountered cell structure or transform into a highly reactive hydroxyl radical (^·^OH) that cannot be neutralized by any of the antioxidants in the human body [45]_._ Despite the observed increase in catalase and peroxidase activity in the parotid glands, cerulein-induced AP downregulates antioxidant capacity of both glands in comparison to the controls. This is indicated by a significant decrease in TAC concentration and is most likely the result of increased production of reactive oxygen species (↑ROS, ↑NOX, ↑XO, and ↑TOS in both salivary glands), which exceeds the antioxidant capacity of the salivary glands. It is worth noting that the decrease in antioxidant defense parameters is greater in the submandibular glands (TAC ↓23%) than in the parotid glands (↓18%) of AP rats. Equally noteworthy is the fact that the main salivary enzymatic antioxidant is peroxidase—the only antioxidant enzyme produced exclusively in the salivary glands [46]. Its increased activity in the parotid glands may be, to some extent, an adaptive mechanism of this salivary gland in response to excessive ROS production, and an insignificant decrease/no change in its activity in the submandibular glands may indicate that these glands are themselves inefficient in combating ROS or do not attempt at serving this function. What is more, the positive correlation between TAC concentration and the activity of salivary peroxidase in the parotid glands suggests that the parotid antioxidant barrier in the course of cerulein-induced AP is maintained mainly by peroxidase activity.

Considering the short time between AP induction and the killing of animals and collection of their tissue (24 hours), in our study, we assessed oxidative stress by measuring only the concentration of protein oxidation products. It was well documented that the protein oxidation occurred as an early event, while lipid peroxidation as a late event in experimental acute pancreatitis [47]. Moreover, the analysis of protein, lipid, and DNA oxidation sequences showed that proteins are the first and almost immediate primary target of ROS attacks, while lipids and DNA are macromolecules effectively protected by proteins and are subject to delayed oxidative modifications [48]. As expected, we observed oxidative damage to the both types of the salivary glands as a significant increase in AGE and AOPP concentration vs. the controls. It should be underlined, however, that the severity of oxidative protein damage was more pronounced in the submandibular glands (AOPP ↑292%) than in the parotid glands (↑130.6%) of AP rats. The presented experiment does not explain a different reaction of the salivary glands to excess free radicals in the course of AP. As our previous studies had demonstrated, the parotid glands are able to provide more efficient antioxidant response than the submandibular glands and have more efficient systems to repair oxidative damage [14, 16, 17, 49, 50].

The negative correlation between AOPP concentration and the level of reduced glutathione in the parotid glands as well as the negative correlation between AGE and reduced glutathione in the submandibular glands of rats from the AP group suggests that increased oxidative stress and oxidative modifications are the result of glutathione deficiency (↓GSH concentration in both glands), which had been explained in the earlier studies. It was proven that the decrease of pancreatic glutathione was involved in the early phase of AP, and the size of this deficit was directly proportional to the extent of the pancreatic injury [51]. Moreover, the positive correlation between TAC concentration and the activity of glutathione reductase in the parotid and submandibular glands in our study suggests that the inefficiency of antioxidant systems of the salivary glands in the early stage of acute pancreatitis is mainly caused by a deficiency of glutathione.

No obvious difference in the secretory response of the salivary glands under no stimulation and after stimulation, 24 hours after the administration of cerulein, may be explained by the work by Marcinkiewicz et al. [52]. The authors showed that the administration of secretin plus cerulein resulted in a significant decrease of saliva secretion during the first 10 minutes, reaching a maximum effect after 20 minutes of continuous infusion. This decreased salivary secretion was maintained throughout the remaining infusion period, and significant differences were also observed after 40 minutes of infusion, returning to normal values one hour after the end of the drug administration. The results by Marcinkiewicz et al. [52] suggest that the interference of cerulein in the mechanisms of water secretion is short term and therefore reversible. Loguercio et al. [53] argue that the reduced saliva secretion, induced by cerulein, could result from it causing contraction of the smooth muscles of the salivary ducts. However, we noticed that—in contrast to the fluid secretion—the mechanism responsible for the synthesis/secretion of proteins appears to be more sensitive to the effects induced by cerulein AP. 24 hours after cerulein administration, the concentration of protein was significantly decreased in the homogenates of both types of the salivary glands of AP rats vs. the control. The lack of morphological changes in the histological study of the salivary glands in connection with the results discussed below may suggest the existence of disturbances at the cellular level. The negative correlation between protein concentrations in the both salivary gland types and salivary amylase activity as well as increased activity of the later in homogenates of both salivary glands may indicate that, similar to in the pancreas, the salivary glands also reveal imbalance of mechanisms inhibiting or stabilizing the activity of enzymes in alveolar cells, which leads to the activation of amylase and other enzymes (HEX, GLU) already within cells of these glands. The increase in the enzymes activities, similar to Sjögren's syndrome [54], could disturb the stromal tissue including a decrease in the number of nerve fibers and the same result in the destruction of neural innervation of the residual acini or adrenergic receptors on the glandular cells and thus decreased response of the glands to the sympathetic stimuli and reduction in protein synthesis/secretion. Selective dysfunction of receptors in the salivary glands was described in the experimental diabetes mellitus. Muscarine receptors in the salivary glands of rats with streptozotocin-induced diabetes have been shown to have reduced susceptibility to acetylcholine as compared to control animals; however, no such changes have been reported to adrenergic receptors [55].

Moreover, the thought that no observed changes in the caspase-3 activity with the coexisting dysfunction of the salivary glands in the course of AP (↓protein concentration) could be the proof that ROS are elevated at moderate level, which is enough to disturb the normal cell function thought it is not severe enough to lead the cell death.

Analyzing the presented results, it should be remembered that our experiment had certain limitations including the animal model, duration of the experiment, and an evaluation of only some biomarkers of redox homeostasis and oxidative stress. On the other hand, we would like to emphasize that the presented analysis of the redox balance and salivary gland function in the course of cerulein-induced AP is a novel and has an important clinical aspect.

## 5. Conclusions

1. Cerlulein-induced AP intensifies production of free radicals, impairs the redox balance of the salivary glands, and is responsible for higher oxidative damage to these glands. Interestingly, oxidative modification of proteins and dysfunction of the antioxidant barrier are more pronounced in the submandibular glands of AP rats
2. Cerlulein-induced AP also disrupts the mechanisms of protein synthesis/secretion in the salivary glands of rats; however, it does not cause morphological changes in the histological examination

## Figures and Tables

**Table 1:** General characteristic of control and AP rats.

	C *n* = 9	AP *n* = 9	*p*
Body weight (g)	272 ± 1.535	261.1 ± 4.48	0.0308
Amylase (IU)	1232 ± 55.2	2321 ± 152.2	<0.0001
Lipase (IU)	130.2 ± 13.1	869.3 ± 110.1	<0.0001
CRP (mg/L)	10.31 ± 3.5	45.2 ± 11.7	0.001
Glucose (mg/dL)	107.7 ± 10.67	133.7 ± 4.385	0.0437
PG weight (g)	87.83 ± 4.318	82.69 ± 3.782	0.3879
SMG weight (g)	219.6 ± 7.67	206.1 ± 9.413	0.2865
Mortality (*n*)	0	1	—

C: controls; AP: acute pancreatitis; CRP: C-reactive protein; PG: parotid glands; SMG: submandibular glands.

**Table 2:** The effect of cerulein AP on serum and plasma redox markers.

	C *n* = 9	AP *n* = 9	*p*
*Enzymatic and nonenzymatic antioxidants*			
SOD serum (mU/mg protein)	4.457 ± 0.8291	2.814 ± 0.5526	0.1215
CAT serum (nmol H_2_O_2_/min/mg protein)	1.103 ± 0.1172	1.532 ± 0.1456	0.0375
GPx serum (mU/mg protein)	0.1063 ± 0.009871	0.07513 ± 0.005437	0.0150
GR serum (uU/mg protein)	30.48 ± 3.056	22.02 ± 0.6425	0.0169
GSH plasma (ng/mg protein)	2.316 ± 0.2375	1.54 ± 0.1185	0.0111
*Redox status*			
TAC plasma (Trolox *μ*mol/mg protein)	0.8814 ± 0.02703	0.6953 ± 0.01159	<0.0001
TOS plasma (nmol H_2_O_2_ Equiv/min/mg protein)	3.757 ± 0.4319	4.79 ± 0.7327	0.2447
OSI plasma	4.315 ± 0.5528	6.895 ± 1.074	0.0508
*Free radical generation*			
NOX plasma (nmol O_2_^−^/min/mg protein)	13.46 ± 0.47	17.75 ± 0.49	<0.0001
XO plasma (*μ*U/mg protein)	21.42 ± 0.75	28.77 ± 0.95	<0.0001
ROS production plasma (nmol/h/mg protein)	10.12 ± 0.11	15.4 ± 0.18	<0.0001
*Protein oxidative damage*			
AGE plasma (AFU/mg protein)	5.227 ± 0.1993	6.197 ± 0.308	0.0192
AOPP plasma (nmol/mg protein)	2.799 ± 0.8918	23.98 ± 4.132	0.0002

C: controls; AP: acute pancreatitis; SOD: superoxide dismutase; CAT: catalase; GPx: glutathione peroxidase; GR: glutathione reductase; GSH: reduced glutathione; TAC: total antioxidant capacity; TOS: total oxidant status; OSI: oxidative status index; AGE: advanced glycation end products; AOPP: advanced oxidation protein products; NOX: NADPH oxidase; XO: xanthine oxidase; ROS: reactive oxygen species.

**Table 3:** The effect of cerulein-induced AP on serum lysosomal exoglycosidases.

	C *n* = 9	AP *n* = 9	*p*
HEX serum (nKat/mg protein)	5.7 ± 0.212	5.74 ± 0.202	0.857
GLU serum (nKat/mg protein)	4.21 ± 0.12	4.52 ± 0.16	0.558

C: control; AP: acute pancreatitis; N-acetyl-*β*-hexosaminidase: HEX; GLU: *β*-glucuronidase.

## Data Availability

The data used to support the findings of this study are included within the article.
